# Among the Colleges

**Published:** 1896-05

**Authors:** 


### AMONG THE COLLEGES.

THE board of regents governing the educational institutions of New York State, acting under State charters, make curious provisions regulating the election of trustees of these institutions. Colleges calling for alumni trustees are asked to act through their alumni bodies and elect from their number those whom they think best fitted for these positions and who have the best interests of their alma mater at heart, and then the names of those elected are subject to acceptance or rejection by the board of trustees elect at their will and pleasure. At the same time they usurp the right to delegate one of the faculty to present a ticket of selected men and instruct the graduating class who are about to become members of the alumni association to vote for these selected candidates, which savors so strongly of a farce that it needs an explanation and light from the board of regents.

The announcement is out for the Kansas City Veterinary College for the obligatory three sessions of six months each, commencing with the term in October next.

The United States Veterinary College of Washington, D. C., again returns to the rôle of three-year colleges with the session of '96 and '97, commencing in October next.

We understand from reliable sources that the National Veterinary College of Washington, D. C., announced in connection with the closing exercises, that the three-term curriculum would be adopted with the opening session of 1896–97.

With the close of the present session of the Veterinary Department of the Detroit College, Professors S. Brenton and McEachran sever their connection therewith. The reasons assigned are differences of views in regard to the future management of the school and the lack of prospect of the Department to extend the curriculum to one of three years of six months each.

The reunion of the alumni of the American Veterinary College, on commencement day, March 25, 1896, brought together a larger representation of the various classes than for several years. Among the older graduates whose presence was noted were Dougherty, '76, Howard, '82, Lowe, '88, Holden, '90, Hoskins, '81, Coates, '77, Neher, '87, Dougherty, '81, Allen, '89, Hanson, '89, and many of those of the more recent classes, together with some twenty-four of the graduating class of 1896.

The meeting proved a very pleasant and enjoyable one, and the selection of Dr. L. H. Howard, of Boston, class of '82, as President, and Dr. John B. Hopper, '92, of Garfield, N. J., as Vice-President, were well-earned honors. The selection of Dr. Otto Faust, class of '88, as Secretary, places this important office in the hands of one fully capable to discharge its arduous duties, and the members can rest assured they will be well done, Dr. Faust, the son of Dr. John Faust, of Poughkeepsie, N. Y., comes from the strongest Association stock, and the example of his father, as an earnest, steadfast Association member and honorable professional associate, will no doubt bear rich fruit through the son in building up and strengthening of the latter's alma mater. The retention of Dr. F. R. Hanson, class of '95, as Treasurer, assures the same fidelity that the Association has enjoyed in the past.

The election of alumni trustees was interesting and spirited, and for the first time selections from outside of the State of New York were conceded to be legal, and for the first time the alumni were informed that their selections and election to these posts of honor and great responsibility were subject to the approval or rejection of the regular board of trustees. For this reason there was submitted by one of the adjunct-faculty three names which the board of trustees desired elected, but, as at least two of these were strangers to the alumni meetings, in deference to the feelings and deep interest of the children in their college parent, other names were placed in nomination, which resulted in the election of but one of the names submitted. Drs. Wm. H. Lowe, Chas. Burden, and L. H. Howard are the three selected, and they are subject to rejection or approval by the board, which may end in the alumni being left without a representative.

Interesting reports were read from several State secretaries, and one of especial interest and pleasure from Dr. Wm. H. Wray, class of '78, of London, Eng., an inspector at that port, under the Bureau of Animal Industry.

Revision of the by-laws in many directions was considered, and other changes suggested left over for another year to complete. The report of the committee for procuring alumni prize was received and approved, and the committee in charge of the banquet reported the completion of their work and announced a number of invited guests. After many pleasant renewals of college experiences and the recalling of pleasant memories within alma mater's walls, and the sad duty of adopting suitable resolutions of regret at the deaths of Drs. R. A. McLean, class of 1879, John Duane, class of 1881, P. F. Kiernan, '87, W. E. Smith, class of 1891, the meeting adjourned to reconvene at Clark's on West Twenty-second Street, at 10.30 P.M., and conclude around the banquet table the festivities of the occasion.

